# Effects of Small and Normalized Q-Factor Changes and Knee Alignment on Knee Biomechanics during Stationary Cycling

**DOI:** 10.3390/bioengineering11090879

**Published:** 2024-08-30

**Authors:** Jacob Wilbert, Sean Brown, Joshua T. Weinhandl, Rachel Tatarski, Songning Zhang

**Affiliations:** Department of Kinesiology, Recreation and Sport Studies, The University of Tennessee-Knoxville, 1914 Andy Holt Avenue, Knoxville, TN 37996, USA

**Keywords:** inter-pedal distance, mechanical axis angle, knee abduction moment, kinetics, lower limb alignment

## Abstract

Increasing inter-pedal distance (Q-Factor: QF) in cycling increases peak internal knee abduction moments (KAbM). The effect of smaller and normalized changes in QF has not been investigated. The purposes of this study were to examine changes in KAbM with small and normalized increases and whether static knee alignment accounts for any changes in knee biomechanics in cycling. Fifteen healthy participants were included (age: 22.7 ± 2.5 years, BMI: 23.95 ± 3.21 kg/m^2^). Motion capture and instrumented pedals collected kinematic and pedal reaction force (PRF) data, respectively, while participants cycled at five different QFs. Each participant’s mechanical axis angle (MAA) was estimated using motion capture. Each participant’s QFs were normalized by starting at 160 mm and increasing by 2% of the participant’s leg length (L), where the five QF conditions were as follows: QF1 (160), QF2 (160 + 0.02 × L), QF3 (160 + 0.04 × L), QF4 (160 + 0.06 × L), and QF5 (160 + 0.08 × L). A linear mixed model was performed to detect differences between QF conditions. KAbM increased by more than 30% in QF5 from QF1, QF2, QF3, and QF4. Medial PRF increased by at least 20% in QF5 from QF1, QF2, and QF3. MAA had varying degrees of correlation with the variables of interest. These results suggest that KAbM is more sensitive to changes in QF at greater QF increases.

## 1. Introduction

Stationary cycling is a low-impact form of aerobic exercise that has been shown to reduce the symptoms of knee osteoarthritis (OA) [1,2,3]. Individuals with knee OA may find stationary cycling preferable for aerobic exercise because the seat supports most of the body’s weight, causing the knee to be relatively unloaded compared to walking [4]. Internal knee extension moment and internal knee abduction moment (KAbM) are commonly used as surrogate variables for total and medial tibiofemoral contact forces, respectively [5,6]. As knee OA affects the medial compartment more commonly than the lateral [7], the objective of knee OA-related biomechanics research is often to reduce KAbM [8].

Increased step width, often evaluated as a percent of an individual’s leg length, is a gait modification that has been shown to reduce KAbM [9,10]. Analogous to step width in gait, the Q-Factor (QF) of a bicycle influences the mediolateral position of the feet, measured as the horizontal distance between the lateral surface of each crank arm, which may affect the frontal-plane knee angles and moments and, potentially, the MCF as a result. However, greater QFs increased KAbM [11] and MCF [12], indicating less preferable loading for individuals with medial compartment knee OA. In the 2020 study by Thorsen et al. [11], QF was incrementally increased by 42 mm, the width of one commercial pedal extender on each pedal, from 150 mm to 276 mm, resulting in increased KAbM with each increase of QF. However, knee extension moment [11] and total tibiofemoral contact force [12] showed no significant differences between QF conditions, and KAbMs were still much smaller than walking [11,13]. While evidence-based guidelines recommend aerobic exercises, including walking, people with knee OA often experience knee pain during weight-bearing exercises, which discourages them from engaging in exercise and can lead to weight gain [2]. This vicious cycle may eventually lead to end-stage knee OA requiring total knee arthroplasty. It is important for people with knee OA to participate in aerobic exercise, and cycling is ideal for that. Normal physiological loading is necessary to protect cartilage health and lead to a lower incidence of knee OA [14,15,16]. It may be beneficial to incorporate changes in QFs into rehabilitation or exercise prescriptions for people with knee OA and those at high risk of developing knee OA. Currently, the optimal loading range to knee medial compartment has not been established. It is possible that some variations of loading level under “the normal physiological loading level” may be beneficial for this patient group. Information on the relationship between QF sizes and levels of KAbM is important for exercise prescription but not well established. Cycling with manipulation of QF provides a perfect experimental model for examining this relationship. Specifically, the sensitivity of KAbM to smaller and normalized changes in QF is unknown.

Static lower limb alignment influences the mediolateral position of the knee relative to the placement of the foot, which may also affect frontal-plane knee angles and moments. This alignment is determined by the orientations of the weight-bearing mechanical axes of the femur and tibia. If the medial angle formed by the intersection of these two axes, known as the mechanical axis angle (MAA), is ≤178°, between 178° and 182°, or ≥182°, the alignments are considered to be varus, neutral, and valgus, respectively [17]. These axes and angles are most accurately determined using full-limb, standing radiography, but hip-knee-ankle angles obtained with three-dimensional (3D) motion capture have been shown to have a high correlation with radiographic MAAs [18]. Greater KAbM and peak knee adduction angles [19] as well as peak tibiofemoral contact force [20] have been seen in individuals with varus alignment during walking, compared to those with neutral and valgus alignments. This effect was also recently investigated during cycling [21]. It was found that peak knee adduction angle, but not KAbM, was significantly greater in varus participants compared to neutral and valgus participants. It was also noted in this study and a study by Fang et al. [22] that participants exhibited either a KAbM or an internal knee adduction moment during the power phase of cycling, with relatively small magnitudes. Shen et al. [21] suggest that knee alignment may be responsible in part for this observation.

To our knowledge, there have been no previous studies that investigate the effect of QF changes less than 42 mm on KAbM, and the sensitivity of KAbM to smaller, normalized changes (relative to leg length) in QF is presently unknown. Disley et al. [23] suggested that the size of QF ranges from 150 mm on a road bike to 180 mm on a mountain bike. The QF for upright cycle ergometers ranges from 147 mm (Wahoo smart bike) and 150 mm (Lode) to 187 mm (Monark) and 209 mm (Kettler). This study used a cycle ergometer with one of the smallest QF, which allowed us to manipulate and increase QF to a level that is still within the upper QF limit of commercial cycle ergometers. Additionally, there have been no studies that investigate static knee alignment as a covariate in the effects of QF on frontal-plane knee moments during ergometer cycling. It is unknown whether static knee alignment will account for any variation in KAbM at different QFs. Therefore, the primary purpose of this study was to investigate whether significant changes in KAbM are detectable with smaller and normalized changes in QF. The secondary purpose of this study was to investigate the relationship between static knee alignment and KAbM. It was hypothesized that KAbM would be greater with each normalized increase in QF. It was also hypothesized that the increases in KAbM would be even greater for people with more varus alignment.

## 2. Materials and Methods

Fifteen adults between 18 and 35 years of age (7 Males, 8 Females, BMI: 23.95 ± 3.21 kg/m^2^) participated in this study. All participants were physically active, free from lower extremity injury within the past 6 months, and free from any history of musculoskeletal disease or lower extremity injury requiring surgical intervention. The minimum sample size was estimated as two using the partial eta squared (η^2^ = 0.721) of a QF main effect on peak KAbM [11] with an α-level of 0.05 and power of 0.80 (3.1.9.7, GPower). A post hoc power analysis confirmed that the study was adequately powered. A written informed consent, approved by the University Institutional Review Board (protocol code UTK IRB-21-06704-XP, 5 January 2022), was signed by each participant prior to testing.

Individual anatomical reflective markers were placed bilaterally on the acromion processes, iliac crests, greater trochanters, lateral and medial femoral epicondyles, lateral and medial malleoli, and the first and fifth heads of the metatarsals. Clusters of four reflective markers were affixed posteriorly or lateral-posteriorly to the trunk, thighs, shanks, and shoes. Five reflective markers were placed on the lateral and anterior sides of each pedal and one marker on each crank axle. Three additional markers were placed on the anterior cover of the ergometer to help track the crank axle’s markers virtually during the movement trials. A static trial was taken with the participant standing on a standardized foot map with the feet oriented anteroposteriorly and separated at the width of the anterior superior iliac spines, which was measured using a caliper, with the stance width measured between the midlines (from heel to 2nd toe) of the feet. The standardized foot map has the lines drawn at known distances from the centerline and was used to standardize the stance position of participants in the static trial, which was designed to improve consistency of MAA estimation from the static trial. A 13-camera motion capture system (240 Hz; Vicon Motion Analysis Inc., Oxford, UK) was used to capture the 3D coordinates of the markers. Pedal reaction force (PRF) was measured using a pair of instrumented pedals, each equipped with two triaxial piezoelectric force transducers (1200 Hz, Type 9027C, Kistler, Winterthur, Switzerland) [11,22,24]. The calibration, verifications, and linearity of the pedals were previously reported [24].

**Figure 1:**
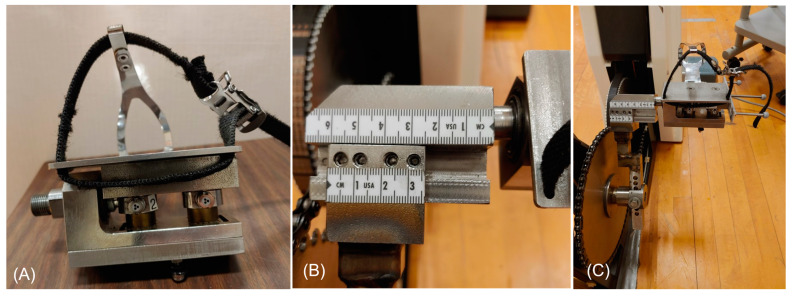
Images of the custom pedal and crank arm assembly. (**A**) Posterior view of the right custom force-instrumented pedal. (**B**) Custom crank arm pedal mount with large mounting block. (**C**) Custom crank arm with reflective markers on Lode ergometer.

**Figure 2:**
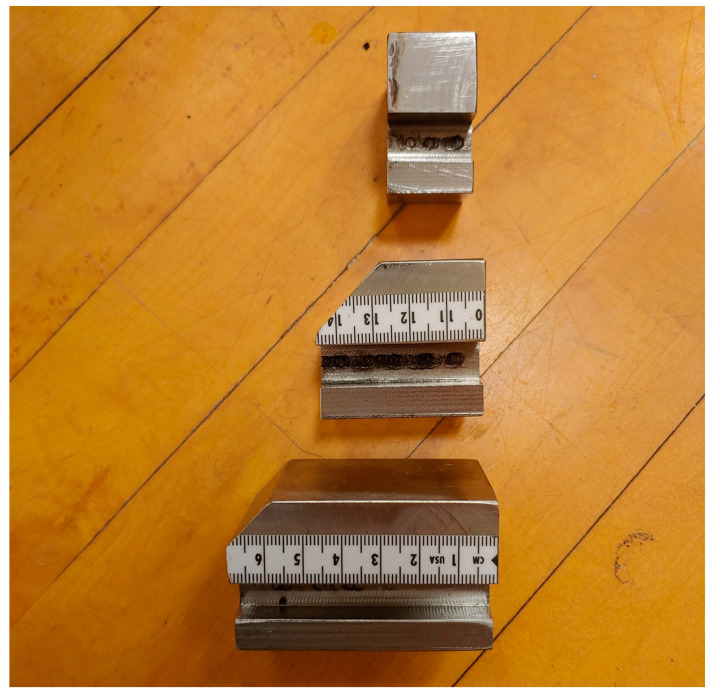
Image of the right small (**top**), medium (**middle**), and large (**bottom**) pedal mounting blocks used with the custom crank arms. The right pedal is screwed into the right face of each block.

Customized adjustable crank arms and a pair of customized instrumented pedals (Figure 1) were used in conjunction with a Lode cycle ergometer (Excalibur Sport, Lode, Groningen, Netherlands) during data collection. The adjustable crank arms allowed for changes in QF using three different-sized blocks (small, medium, large; Figure 2) for mounting the pedals onto the crank arms to create a desired QF. Any conditions with a QF at or below 172 mm were achieved using the small block, a QF between 173 mm and 232 mm was achieved using the medium block, and a QF at or above 233 mm was achieved using the large block. The saddle height was adjusted so that the knee angle was between 25° and 30° when the pedal was at the bottom dead center [25,26]. The saddle fore-aft position was set where the participant’s knee was aligned vertically with the pedal spindle when the crank arm was at its most forward position. The position of the handlebar was set where the angle between the trunk and thigh was 90° when the pedal was at its most forward position. The feet were strapped onto the pedals using a toe-cage.

After a two-minute warmup, each participant completed five QF conditions: QF1 (160 mm), QF2 (160 mm + 0.02 × L), QF3 (160 mm + 0.04 × L), QF4 (160 mm + 0.06 × L), and QF5 (160 mm + 0.08 × L), where L is leg length, measured in a standing position from greater trochanter to floor, and each condition was changed by two percent of L. The QF conditions were randomized in two different steps. Since the desired personalized QFs for each participant are achieved through a combination of two or three pedal mounting blocks, the order of pedal mounting block sizes was first randomized, and the order of the QF conditions that can be achieved within each mounting block was then randomized. The average QF change between conditions for all participants was 18 mm. Each condition was performed for two minutes at a work rate of 120 Watts and a cadence of 80 revolutions per minute. Kinematics and kinetics during the final 10 s of each condition were recorded [26]. Participants were given at least two minutes of rest between conditions. The 10 s of dynamic trial data were cropped to five consecutive crank cycles for analyses. A crank cycle was defined as a full revolution of the crank arm with the beginning (0°) and end (360°) at the top dead center (TDC) position.

Kinematic and kinetic computations were performed in Visual3D (Version 6, C-Motion Inc., Germantown, MD, USA). Marker coordinate data and analog pedal reaction force data were filtered using a fourth-order, zero-lag, low-pass Butterworth filter with a cutoff frequency of 6 Hz [26]. Angular kinematics and kinetics were computed using the joint coordinate system [27] and an XYZ Cardan sequence and expressed using the right-hand rule. Joint moments were computed using an inverse dynamics approach and expressed as the internal moments and reported in the proximal segment. Limb inertial characteristics were estimated using a combination of the Demspter [28] and Hanavan [29] anthropometric models in Visual 3D. The hip joint center location was estimated using an offset of 23.4% medially and 4.7% superiorly of the intertrochanteric distance from the ipsilateral greater trochanter marker location [30]. Sagittal and frontal-plane ranges of motion (ROM, calculated as the difference between values at onset and minimum/maximum), peak angles, and peak moments were calculated for each participant’s dominant side (the leg with which they would kick a soccer ball). The hip-knee-ankle (HKA) angle was calculated and expressed as its deviation from 180° (HKA deviation = HKA − 180°; adduction = negative; abduction = positive) to estimate the mechanical axis angle (MAA) based on Equation (1) [18].

MAA = (−4.05 + 1.05 × HKA deviation) + 180°(1)

A Shapiro–Wilk test was used to test the data for normality. The results showed that the examined variables were normally distributed. Pearson correlation coefficients were computed between MAA and variables of interest. Linear mixed models, using a compound symmetry covariance structure, were used to investigate within-subject differences between QF conditions in the variables of interest and included MAA as a covariate. It was found that MAA did not have any meaningful effects on the key loading variables; therefore, it was removed from the final models. If a main effect of QF was detected, post hoc pairwise comparisons between conditions were made with Bonferroni adjustments. Analyses were run using IBM SPSS 28, Chicago, IL, USA. The α-level was set at 0.05 a priori.

## 3. Results

**Figure 3:**
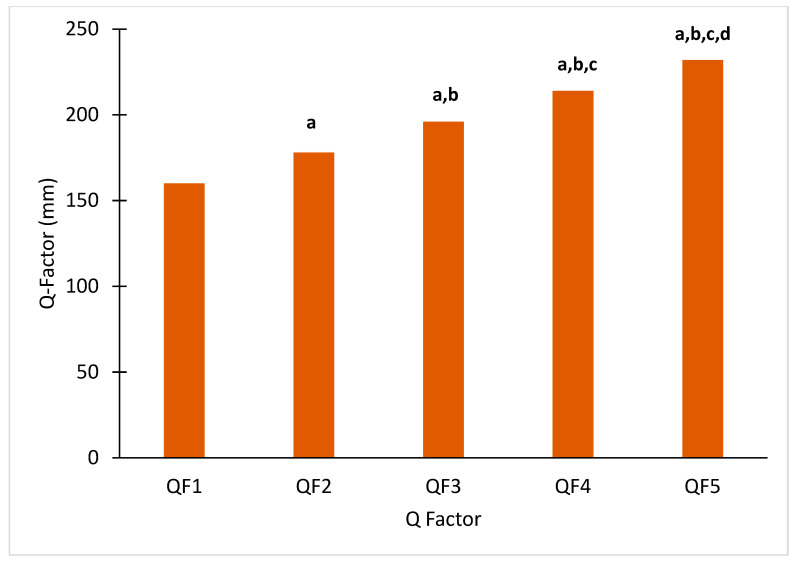
Mean QF values for QF1 to QF5. Significantly different compared to ^a^ QF1, ^b^ QF2, ^c^ QF3, ^d^ QF4.

The width of each QF condition was significantly different from each of the other QFs (*p* < 0.001 for all pairwise comparisons, Table 1 and Figure 3). The mean MAA value was 175.5° (±2.3°) with a range between 172.3° and 179.7°. Among the participants, 12 had a varus alignment (<178°) and three a neutral (≥178° and ≤182°) alignment.

The ANOVA results found a significant QF effect on peak vertical PRF (*p* = 0.01, Table 1), and post hoc comparisons showed that vertical PRF was significantly greater for QF1 than QF2 and QF4 (*p* ≤ 0.017 for both comparisons). The QF main effect was also significant for peak medial PRF (*p* < 0.001, Table 1). The post hoc comparisons found that it was significantly greater (more negative) in QF5 than in QF1, QF2, and QF3 (*p* < 0.001 for all comparisons).

**Figure 4:**
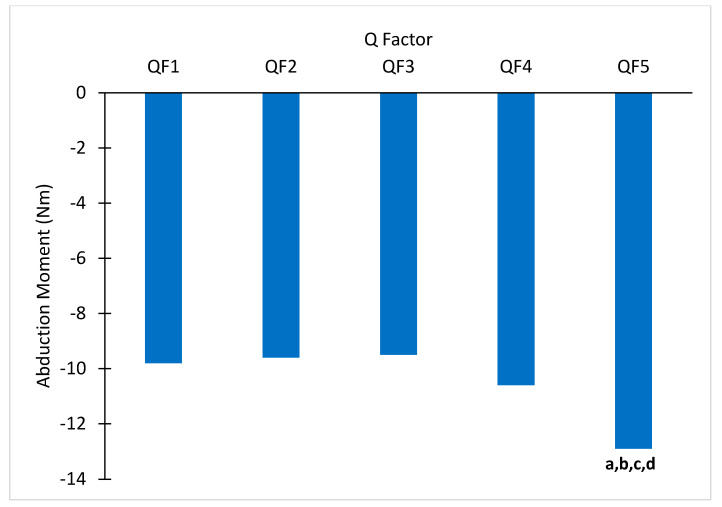
Mean peak knee abduction moments across five QFs. Significantly different compared to ^a^ QF1, ^b^ QF2, ^c^ QF3, ^d^ QF4.

There was a significant main effect of QF on peak KAbM (*p* < 0.001, Table 1 and Figure 4), and the post hoc comparisons found the KAbM was significantly greater for QF5 than QF1, QF2, QF3, and QF4 (*p* ≤ 0.004 for all comparisons). There was a significant QF main effect on knee abduction ROM (*p* <0.001, Table 1). The post hoc analyses found that it was significantly smaller for QF4 than QF1 (*p* = 0.009) and smaller for QF5 than QF1 and QF3 (*p* ≤ 0.008 for both comparisons).

There was significant correlation found between MAA and vertical PRF at QF4 only (r = −0.561, *p* = 0.03; Table 2) as well as between MAA and knee abduction ROM at QF5 only (r = −0.580, *p* = 0.038).

## 4. Discussion

The main purpose of this study was to investigate whether significant changes in KAbM are detectable with smaller and normalized changes of QF. The primary hypothesis that KAbM would become greater with increased QFs was partially supported by the results of this study. Although each increase from one QF to the next was equal in magnitude, not all changes in QF resulted in significantly increased KAbM. Interestingly, the peak KAbM in QF1 through QF4 was not statistically different, but the peak KAbM for QF5 was statistically different from all other conditions. With a mean QF difference (2% of leg length) of 18 mm between conditions across all participants, these comparisons equated to average differences in QF of 18 mm (QF4–QF5; range: 16–20 mm), 36 mm (QF3–QF5; range 32–40 mm), 54 mm (QF2–QF5; range 48–60 mm), and 72 mm (QF1–QF5; range 64–80 mm). These increments are smaller than what has been shown to have a significant effect on KAbM in previous literature (42 mm) [11]. The most relevant of these comparisons is the significant difference between QF4 and QF5, where peak KAbM increased by 21.7%. In terms of absolute QF change, the difference between QF4 and QF5 is a considerably smaller difference in QF than has previously been shown to cause significant increases in KAbM. Thorsen et al. [11] used 42 mm increments in QF at the same work rate (120 Watts) and cadence (80 revolutions per minute) as the present study and found significant increases in KAbM with each incremental increase in QF from 150 mm to 276 mm. The results of the present study indicate that it is possible to see a significant increase in KAbM with as small of a change as 2% of leg length, which ranged from 16 to 20 mm for the participants in this study. However, the fact that other QF changes of equal and greater magnitude did not cause significant changes in KAbM should not be ignored.

It was expected that if a single incremental increase in QF caused an increase in KAbM, then the other changes of the same and greater magnitude would also cause increases in KAbM. A potential explanation for why this was not observed may be that the relationship is nonlinear, where the change in KAbM is greater at higher QFs. This is supported by the present findings that KAbM at QF5 was significantly higher than at QF4 and the other three QFs, while there were no significant differences between QF1, QF2, QF3, and QF4 themselves.

The PRF can influence KAbM in different ways. The medial PRF is more influential in modulating the length of the frontal-plane moment arm of the knee, while the vertical PRF has a greater influence on the magnitude of the resultant PRF vector. The medial PRF was significantly increased in QF5 compared to QF1, QF2, and QF3 in the present study. A general increase in medial PRF with increased QFs was expected and agrees with previous literature [11]. There were a couple of significant differences found in vertical PRF, but they did not likely have a meaningful influence on the observed differences in KAbMs. These differences in vertical PRF were not observed concomitantly with significant changes in KAbM. Additionally, there was no consistent pattern in these changes, which is supported by previous research [11]. Given a constant vertical PRF between QF5 and the other conditions, it appears that the increases in KAbM were primarily caused by increases in the PRF moment arm and, to a lesser extent, by the PRF itself. In most comparisons, this is supported by either concurrent increases or lack thereof in both medial PRF and KAbM. The only comparison where this was not upheld was between QF4 and QF5, where there was a significant increase in KAbM but not in medial PRF. This could be explained by the fact that there are other variables that can influence KAbM, such as the frontal-plane knee angle, that may have contributed to this change.

The frontal-plane knee angle can also influence KAbM by changing the position of the knee relative to the PRF, thereby changing the length of the moment arm. The most common pattern of knee frontal-plane angle during the early power phase was knee abduction among our participants. Peak knee abduction angle occurred almost simultaneously with the peak KAbM, but no differences in peak knee abduction angles between conditions were found. Thorsen et al. [11] and Fife et al. [31] also investigated the effect of QF on peak knee abduction angle, and they both found that, to some extent, increases in QF caused the knee to become more abducted. These findings disagree with the current study, but the greatest change in knee abduction angle in either of these studies was only about 2° between QFs of 150 and 276 mm [11]. We also observed that the knee abduction ROMs in the present study were significantly smaller in QF4 than in QF1 and in QF5 than in QF1 and QF3 (Table 1). The lack of significant changes in peak knee abduction angles indicates that at the onset of the crank cycle, the knee was less adducted in the higher QFs. Given the results of the current study and these studies [11,31], it appears that changes in peak frontal-plane knee angles and their ROMs during the power phase may not have a meaningful contribution to changes in peak KAbM by themselves. It is possible that subtle changes in frontal-plane knee angle, in combination with subtle changes in medial PRF, may result in more notable changes in KAbM. This may explain why there was a significant increase in KAbM, but neither peak knee abduction angle nor medial PRF was significantly different in the same comparison. Additionally, variability in the temporal overlap between the peak medial PRF, vertical PRF, and knee abduction angle may explain the current observations. In order to determine how these variables contribute to changes in peak KAbM, further investigation would need to be performed to determine each variable’s effect on the moment arm at the instance of peak KAbM.

The secondary aim of this study was to examine the relationship between an individual’s MAA and their KAbM. The hypothesis that MAA would account for some variance in KAbM was not supported by the findings of the current study. MAA did not account for any significant portion of the variance in KAbM based on the initial ANCOVA, nor was a significant correlation between the two variables found (|r| ≤ 0.197, *p* ≥ 0.482 for all QF conditions). In a previous study of the effects of knee alignment on knee biomechanics during cycling, no significant effects were found for the knee alignment group on KAbM, mediolateral PRF, or vertical PRF [21]. These alignment group comparisons cannot be directly related to the current study, but they lend support to the findings that there was no consistent correlation between MAA and these variables. There was a significant correlation between MAA and vertical PRF in QF4, but considering the ANCOVA results and lack of significant correlation elsewhere, this relationship is likely not meaningful. The same previous study found that knee alignment significantly affects the peak frontal-plane angles of the knee [21]. The current study found no relationship between MAA and the peak knee abduction angle, although there was a significant correlation between MAA and knee abduction ROM for QF5. Again, these results cannot be directly compared due to differences in participant groups and study aims, but they would seem to disagree about the nature of the relationship between MAA and peak frontal-plane knee angles. Static knee alignments are most accurately determined through standing and full-limb radiography, but the present study used 3D motion capture and a regression model from previous literature to estimate MAA [18]. Differing methods of determining MAA may explain some of the observed differences between the present study and studies that utilized radiography, such as those by Shen et al. [21]

As previously stated, KAbM may be influenced by the vertical PRF, medial PRF, and the frontal-plane knee angle. Since MAA had no consistent relationships with these variables, it is agreeable that the same was found for KAbM. Static frontal-plane knee alignment was expected to influence peak frontal-plane angles of the knee and peak KAbM, as has previously been shown in level walking [19,32]. While walking and cycling share similarities, ultimately, they have different dynamic processes. Previous research has already shown that KAbM is increased by widening the pedals in cycling [11] and decreased by widening the stance in walking [19]. Additionally, MAA is captured in a standing posture, which is a weight-bearing position similar to walking. Conversely, cycling is performed in a seated, non-weight-bearing position where both the feet (by pedals) and hips (by saddle) are more constrained than in walking, leading also to more constrained movement. Therefore, it is reasonable to expect that MAA may affect cycling dynamics differently than it affects walking. However, it cannot be concluded entirely that MAA bears no effect on cycling biomechanics, as the participants in this study had relatively homogenous MAA alignments, and no participants had valgus alignment. Therefore, it remains possible that individuals with valgus alignment respond differently to changes in QF than individuals with varus or neutral alignment.

One limitation is that the crank arm and adjustable QF assembly were custom-built, so it would be difficult for outside research groups to replicate these conditions. The crank arms and pedals were also considerably heavier and bulkier than commercially available parts. It is possible that the increased inertia of the crank arm and pedal assembly could have altered the biomechanics of the rider. This warrants further investigation to examine if the increased inertia contributed to changes in sagittal-plane and frontal-plane lower limb kinetics. Participants in the study are younger, college-aged students, and their responses to the QF changes may be different from those with knee OA who are typically older. It is warranted to examine people with knee OA in the future. Another limitation of this study was that MAA was not determined using radiography but was estimated using a validated 3D motion capture and a regression equation from previous literature [18].

## 5. Conclusions

This study was the first to investigate the effects of small and normalized changes in QF while controlling for static frontal-plane knee alignment. The results of this study show that it is possible to detect a significant increase in KAbM with changes in QF as small as 2% of one’s leg length at a high QF. These findings suggest that KAbM becomes more sensitive to changes in QF at greater QFs. Static knee alignment does not appear to be meaningfully related to any of the knee kinetic variables. The results suggest that people with varus and neutral knee alignment may not need to be concerned about alignment-associated changes in knee loading when cycling at different pedal widths on stationary bikes and cycle ergometers.

## Figures and Tables

**Table 1:** Mean QFs (mm), peak PRFs (N), peak knee angles and ROMs (°), peak knee moments (N m), and mean (SD).

Variables	QF1	QF2	QF3	QF4	QF5	*p*
QF	160.0 (0.0)	178.0 (1.0) ^a^	196.0 (2.0) ^a,b^	214.0 (3.0) ^a,b,c^	232.0 (4.0) ^a,b,c,d^	<0.001
Vertical PRF	233.5 (34.8)	217.4 (32.6) ^a^	222.2 (29.2)	217.2 (27.0) ^a^	224.8 (27.9)	0.010
Medial PRF	−45.3 (10.5)	−45.7 (7.8)	−46.1 (9.5)	−50.1 (10.2)	−55.4 (11.2) ^a,b,c^	<0.001
Extension Moment	34.7 (7.2)	35.2 (4.8)	34.9 (5.6)	35.1 (5.9)	38.1 (6.8)	0.590
Abduction Moment (KAbM)	−9.8 (4.5)	−9.6 (4.1)	−9.5 (4.5)	−10.6 (4.0)	−12.9 (4.6) ^a,b,c,d^	<0.001
Extension Angle	−33.1 (6.4)	−34.0 (6.7)	−33.2 (6.5)	−32.6 (7.0)	−32.6 (6.3)	0.148
Extension ROM	77.4 (7.5)	76.9 (7.4)	76.8 (7.4)	77.7 (7.6)	77.9 (7.3)	0.186
Abduction Angle *	0.83 (4.17)	1.19 (3.99)	0.76 (4.00)	0.48 (4.19)	0.15 (3.93)	0.145
Abduction ROM *	−5.7 (2.6)	−4.9 (2.0)	−5.2 (2.5)	−4.3 (2.7) ^a^	−3.8 (2.5) ^a,c^	<0.001
Adduction Angle **	5.2 (0.5)	5.9 (0.2)	6.0 (0.8)	5.3 (0.2)	5.3 (0.2)	-
Adduction ROM **	1.5 (1.3)	1.5 (1.3)	1.7 (1.2)	2.2 (0.5)	3.7 (0.8)	-

Abbreviations: QF—Q-Factor, PRF—pedal reaction force, ROM—range of motion, KAbM—internal knee abduction moment. Significantly different compared to ^a^ QF1, ^b^ QF2, ^c^ QF3, ^d^ QF4. * 13 out of 15 participants displayed this pattern. Values for peak knee abduction angle refer to the minimum angle closest to an abducted position. ** 2 out of 15 participants displayed this pattern. Statistical procedures were not performed on these variables.

**Table 2:** Pearson correlation coefficients between MAA and selected PRF and knee variables: (*p* value, n—sample size).

Variables	QF1	QF2	QF3	QF4	QF5
Vertical PRF	−0.358 (0.209, 14)	−0.279 (0.313, 15)	−0.455 (0.102), 15)	−0.561 * (0.03, 15)	−0.340 (0.216, 15)
Medial PRF	0.064 (0.828, 14)	0.228 (0.413, 15)	0.419 (0.136, 14)	0.432 (0.108, 15)	0.212 (0.448, 15)
Peak Abduction Moment (KAbM)	−0.055 (0.853, 14)	−0.100 (0.724, 15)	−0.038 (0.898, 14)	−0.197 (0.482, 15)	−0.086 (0.761, 15)
Peak Extension Moment	−0.090 (0.761, 14)	0.073 (0.796, 15)	0.346 (0.226, 14)	0.377 (0.166, 15)	0.210 (0.452, 15)
Peak Abduction Angle	−0.274 (0.364, 13)	−0.348 (0.244, 13)	−0.390 (0.187, 13)	−0.391 (0.186, 13)	−0.418 (0.155, 13)
Knee Abduction ROM	−0.474 (0.102, 13)	−0.374 (0.208, 13)	−0.304 (0.312, 13)	−0.262 (0.387, 13)	−0.580 * (0.038, 13)

Abbreviations: MAA—Mechanical axis angle, PRF—pedal reaction force, ROM—range of motion, KAbM—internal knee abduction moment. *: *p* < 0.05.

## Data Availability

Due to privacy restrictions, the data presented in this study are available upon request from the corresponding author.
